# Tolerogenic Immunotherapy: Targeting DC Surface Receptors to Induce Antigen-Specific Tolerance

**DOI:** 10.3389/fimmu.2021.643240

**Published:** 2021-02-19

**Authors:** Charlotte Castenmiller, Brigitte-Carole Keumatio-Doungtsop, Ronald van Ree, Esther C. de Jong, Yvette van Kooyk

**Affiliations:** ^1^Department of Experimental Immunology, Amsterdam University Medical Centers, Amsterdam Institute for Infection & Immunity, University of Amsterdam, Amsterdam, Netherlands; ^2^Department of Molecular Cell Biology and Immunology, Amsterdam University Medical Centers, Amsterdam Institute for Infection & Immunity, Vrije Universiteit Amsterdam, Amsterdam, Netherlands; ^3^Department of Otorhinolaryngology, Amsterdam University Medical Centers, University of Amsterdam, Amsterdam, Netherlands

**Keywords:** dendritic cell, tolerance, immunotherapy, surface receptors, C-type lectins, Siglecs, allergy, auto immune diseases

## Abstract

Dendritic cells (DCs) are well-established as major players in the regulation of immune responses. They either induce inflammatory or tolerogenic responses, depending on the DC-subtype and stimuli they receive from the local environment. This dual capacity of DCs has raised therapeutic interest for their use to modify immune-activation *via* the generation of tolerogenic DCs (tolDCs). Several compounds such as vitamin D3, retinoic acid, dexamethasone, or IL-10 and TGF-β have shown potency in the induction of tolDCs. However, an increasing interest exists in defining tolerance inducing receptors on DCs for new targeting strategies aimed to develop tolerance inducing immunotherapies, on which we focus particular in this review. Ligation of specific cell surface molecules on DCs can result in antigen presentation to T cells in the presence of inhibitory costimulatory molecules and tolerogenic cytokines, giving rise to regulatory T cells. The combination of factors such as antigen structure and conformation, delivery method, and receptor specificity is of paramount importance. During the last decades, research provided many tools that can specifically target various receptors on DCs to induce a tolerogenic phenotype. Based on advances in the knowledge of pathogen recognition receptor expression profiles in human DC subsets, the most promising cell surface receptors that are currently being explored as possible targets for the induction of tolerance in DCs will be discussed. We also review the different strategies that are being tested to target DC receptors such as antigen-carbohydrate conjugates, antibody-antigen fusion proteins and antigen-adjuvant conjugates.

## Introduction

Dendritic cells (DCs) are important antigen presenting cells during the induction of immune responses and are essential in directing immune responses toward either immunity or tolerance. This decision is of great importance as undesired inflammatory responses could cause autoimmune or allergic diseases. In the periphery, DCs capture antigens and process them while migrating to the draining lymph nodes, where they present antigen-specific peptides to T lymphocytes. This migration process causes a dramatic transformation of the DC phenotype, called maturation. Maturation is associated with increased MHC-II complex levels, costimulatory molecule expression, enhanced secretion of polarizing cytokines and molecules, and alterations in chemokine receptor expression, all resulting in an optimal microenvironment to direct T cell responses (1–3). Although various DC subsets have been shown to preferentially induce specific T cell responses in non-inflammatory conditions, the induction of T cell immunity is adapted to and dictated by the encounter with pathogens (4). The unique capacity of DCs to coordinate innate and adaptive immune responses has highlighted them as potential targets for immune activating or dampening therapies to combat undesired immune responses (3).

**Figure 1:**
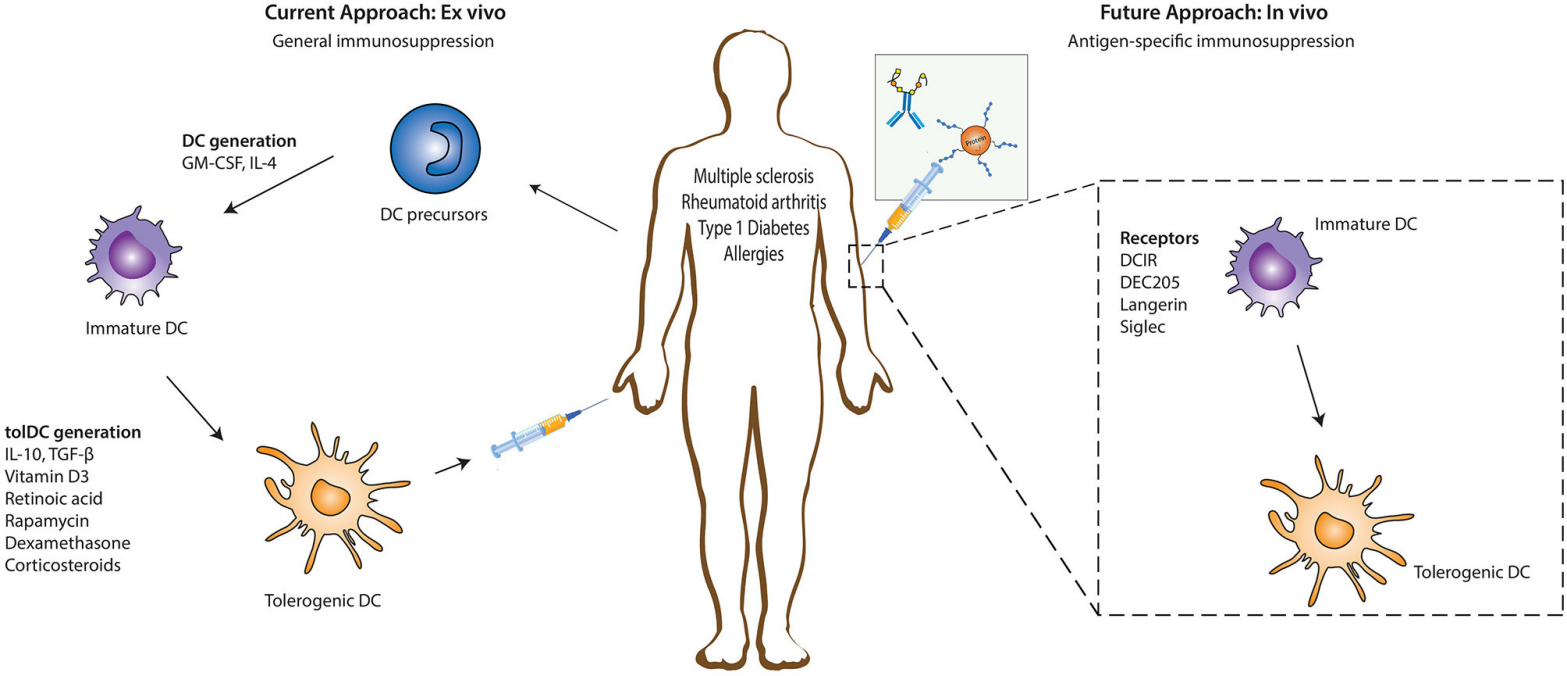
*Ex vivo* and *in vivo* strategies for generation of tolerogenic DCs for DC-based therapies. The current DC-based immunotherapy strategy in the treatment of immunopathologies involves the isolation of DC precursors either from PBMCs or bone marrow-derived cells which could either be allogeneic or autologous. These DC precursors are then differentiated into immature DCs in the presence of GM-CSF and recombinant IL-4 which are subsequently differentiated into tolerogenic DCs (tolDCs) by the addition of pharmacologic agents or immunomodulatory cytokines. Administration of these tolDCs leads to the generation of a suppressive immune environment which dampens inflammation. Future strategies are focusing more on *in vivo* targeting of DCs, where specific antigen-based vaccine formulations targeting specific receptors on DCs in their natural environment are injected into the patient. The antigen is taken up by DCs through these receptors, resulting in the induction of a tolerogenic program in DCs that leads to the generation of antigen-specific immunosuppression. DC, dendritic cells; GM-CSF, Granulocyte-macrophage colony-stimulating factor; IL-4, Interleukin 4; IL-10, Interleukin-10; TGF-β, Transforming growth factor beta.

Immunomodulatory agents such as vitamin D3, retinoic acid, rapamycin, dexamethasone, corticosteroids, ligands of the aryl hydrocarbon receptor (AhR), or specific cytokines (IL-10, TGFβ) have been key in determining the existence and function of tolerogenic DCs (tolDCs) *ex vivo* (Figure 1) (5–7). These tolDCs can induce tolerance through various mechanisms, including the induction of Tregs, autoreactive T cell anergy and apoptosis, and could be used in tolerizing immunotherapies (6, 8, 9). *Ex vivo* tolDC immunotherapies are based on re-education of patient-derived DCs to a tolerizing phenotype and the subsequent reinfusion into the body, where they suppress inflammatory immune responses (Figure 1). The first clinical study utilizing tolerogenic DCs (tolDCs) for the treatment of autoimmune diseases was performed in 2011 in adult type I diabetes (T1D) patients. Since then, phase I and II clinical trials have been conducted for T1D, rheumatoid arthritis (RA), Crohn's disease, and multiple sclerosis (MS) (5), but also for kidney and liver transplant recipients (8–10). However, due to the personalized, laborious, and expensive nature of *ex vivo*-generated tolDCs, new approaches for inducing tolDCs *in vivo* are being developed.

The feasibility and potential of *in vivo* strategies lie in the ability of DCs to recognize and internalize antigens through surface receptors that not only route antigens to the antigen processing machinery of DCs for subsequent presentation to T cells but also transmit signals that direct anti-inflammatory immune responses. This allows direct modulation of specific DC subsets due to differential surface receptor expression profiles between them. *In vivo* DC-targeting has several advantages compared to *ex vivo* DC-targeting, including fewer hospital visits for the patient, less laborious production methods, and the possibility of large scale production, which is more cost-effective. Additionally, the induction of antigen-specific T cell responses with *in vivo* DC-targeting strategies reduces the risk of generalized immunosuppression, which is induced during the current *ex vivo* strategies using only immunosuppressive agents. The main strategies for *in vivo* tolDC generation take advantage of modalities binding to specific endocytic receptors on DC surfaces, ensuring the delivery of antigen of interest into the antigen-processing machinery (Figure 1) (11). Antigens could either be directly coupled to antibodies (11) or loaded on nanoparticles or in liposomes, reviewed elsewhere (12). Another strategy being explored in this regard involves chemically conjugating antigens with specific glycan structures which are ligands for DC surface receptors. In this review we discuss the different DC-subsets used for targeting, the receptors expressed on their surface that have potential to induce tolerogenic signals (but might not be inherently tolerogenic), and the current state of research in their use for the treatment of auto-immune or allergic diseases.

## Human Dendritic Cell Subsets and the Induction of Tolerogenic Dendritic Cells

It is now recognized that DCs are a heterogeneous population of cells. The different subsets are defined by surface markers and transcriptome profiles, nicely reviewed by various colleagues (4, 13–16). DCs are generally classified into four major subsets, namely, CD141^+^ conventional DCs (cDC1s), CD1c^+^ conventional DCs (cDC2s), monocyte-derived DCs (moDCs), and plasmacytoid DCs (pDCs). The cDC1 subset is a relatively homogenous population that is specialized in cross-presentation of extracellular antigens and efficiently primes CD8^+^ T cells (16). In contrast, the cDC2 subset is a heterogeneous population and could be further subdivided in separate lineages. For example, the cDC2A and cDC2B lineage are defined by distinct developmental pathways regulated by the transcription factors T- bet and RORγt, respectively (17). Both lineages are potent stimulators of CD4^+^ naïve T cell, however, cDC2Bs have been shown to be more prone to secrete pro-inflammatory cytokine than cDC2As (13, 17). Additionally identified cDC2 lineages include monocyte-like DC2s, inducing Th1 responses, and DC3s, responsible for Th2, Th17 and Treg differentiation (15, 17). The moDC subset arises from monocytes and retains, like the DC3s, the monocyte marker CD14. They are recruited to inflamed tissue sites *in vivo* where they efficiently cross-present antigens to CD8^+^ T cells in peripheral tissues (18). The last subset, pDCs, differs from the other subsets as they are marked by quick secretion of pro-inflammatory type I interferons (IFN) following viral infection. pDCs are defined as CD123^+^ CD303^+^ CD304^+^ cells and were originally classified within the myeloid compartment. However, recent findings providing evidence for a lymphoid origin of the majority of pDCs challenges this hypothesis (4, 13, 19, 20). Finally, the tissues where DCs reside, such as lymph nodes, skin, lung, intestines and liver, offer the above mentioned DC subsets additional environmental factors to further adapt to their specific niche resulting in tissue specific DC subsets (8, 21, 22).

Although the immune system encounters many innocuous antigens, including self-antigens and allergens, the chance to develop autoimmune or allergic diseases is relatively small due to the phenomenon of “natural tolerance.” Natural tolerance is achieved through the presence of tolerance-inducing DCs located both centrally and in the periphery. Central tolerance induction is mediated by thymic epithelial cells and thymic DCs, which regulate negative selection of autoreactive T cells and induction of natural Tregs (23, 24). Even though the specific role of each thymic DC subset in peripheral immune homeostasis remains elusive, thymic pDCs and the Sirpα^+^ cDC subset have been proposed to contribute to the prevention of allergic or commensal-specific autoimmune diseases, as they originate from the periphery where they encounter many innocuous antigens, followed by migration to the thymus (23–25). On the other hand, peripheral tolerance is mediated by peripheral DCs, preferentially located at the border between the body and the external environment, such as lung, intestine and skin. Steady state or immature DCs were the original identified peripheral tolDCs (26–28). They exhibit low expression of co-stimulatory (CD40, CD80/86) and MHC molecules due to lack of appropriate activation signals and are able to maintain tolerance *via* deletion of self-reactive T cells, induction of T cell anergy or differentiation of antigen-specific Tregs (2). These immature DCs have been reported to be the primary cell types involved in maintaining tolerance in the periphery and mainly carry self-antigens. However, recent identification of partial- or semi-mature DCs with tolerizing capacities, questions the dogma that only immature DCs induce tolerance (22, 27). Similar to immunogenic DCs, tolDCs may be defined by integration of all the signals they transmit to T cells, including maturation marker expression, as well as the presence of, in this case, anti-inflammatory-related tolerizing signals consisting of surface molecule expression (PD-L1, ILT3/4, ICOSL, CTLA-4), tolerogenic cytokine profiles (IL-10, TGFβ) and the presence of other tolerance-inducing metabolites (IDO, RA) (14, 22). Furthermore, the presence or absence of pro-inflammatory cytokines seems to be decisive in inducing either immunity or tolerance, respectively. Nevertheless, no standard tolDCs profile has been established yet, and may not exist due to the great diversity between those that have been described till date.

Besides the immature and semi-mature tolDCs, several tissue-specific DCs exhibit inherent tolerogenic properties, including those in the skin and intestines. In the skin, Langerhans cells (LCs), which are characterized by the expression of Langerin (CD207), CD1a, E-Cadherin, CD39, FcεRI, and Birbeck granules, are the sole tissue-resident DC population in the epidermis (29). LCs constantly migrate from the skin to draining lymph nodes, even in steady-state conditions, and have been implicated in both immunogenic as well as tolerogenic immune reactions (30–32). In contrast, CD14^+^(CD141^+^) dermal DCs constitutively secrete the anti-inflammatory cytokine IL-10 and are prone to induce T cell anergy and Tregs that inhibit skin inflammation (33, 34). The ability to produce extensive levels of IL-10 is shared with CD14^+^CD16^+^CD141^+^CD163^+^ DCs isolated from peripheral blood, identified by Gregori and colleagues, which might correspond to the DC3 subset expressing the same surface markers (16, 35). The same group has shown that these cells express the surface receptors HLA-G, ILT2, ILT3, and ILT4 and have the potency to induce type 1 Tregs (Tr1) *in vitro* (36–38). In the intestines, the main subset involved in oral tolerance during steady state conditions are the CD103^+^ DCs in the lamina propia and mesenteric lymph nodes. They are able to prime Tregs in gut lymphoid tissues through the production of TGF-β and RA (39–42). Additionally, CD103^+^ DCs express high levels of RALDH2, converting vitamin A to RA which enhances Treg induction (29). These studies demonstrate that various tissues contain specific subsets of tolDCs, emphasizing the power of the immune system to adapt to specific environmental factors functioning to maintain immune homeostasis during tissue-specific circumstances.

## Dendritic Cell Pathogen Recognition Receptors Modulating Immune Responses

Signals such as pathogen-associated molecular patterns (PAMPS) from pathogens, damage associated molecular patterns (DAMPS) from inflammation, and self-associated molecular patterns (SAMPS) can be recognized by pattern recognition receptors (PRRs) on the surface of DCs (21, 43). C-type lectins (CLRs) and Sialic-acid binding immunoglobulin-type lectins (Siglecs) are families of PRRs equipped with a carbohydrate recognition domain that specifically recognizes glycan moieties on host cells, pathogens, as well as innocuous antigens such as allergens (21, 43–46).

CLRs function both as adhesion molecules and endocytic receptors, but also have a function in directing immunity to various pathogens, cellular proteins and lipids (44, 47, 48). Induction of immune response through these receptors can alternate between inflammation and immune tolerance depending on several factors including the nature of the ligand (49). They recognize a large and diverse range of ligands and trigger immune responses by inducing signaling pathways *via* an immunoreceptor tyrosine-based activation motif (ITAM), ITAM-like motif, or immunoreceptor tyrosine-based inhibitory motif (ITIM) that signal through Syk or phosphatases (21, 44, 45, 49, 50), generating pro- or anti-inflammatory signals, respectively. Only a few CLRs, such as, DC immunoreceptor (DCIR), Clec12A and Clec12B, bear the ITIM motif (51). Moreover, an important number of CLRs do not signal through Syk or phosphatases but may bear ITAM/ITIM motifs which are important for endocytosis (45, 52). Examples include: dendritic cell-specific intercellular adhesion molecule-3-grabbing non-integrin (DC-SIGN), LSECtin, macrophage C-type lectin (MCL), Langerin, macrophage-galactose lectin (MGL), mannose receptor (MR) and DEC205 (11, 44, 48). These CLRs mediate antigen internalization, followed by processing and subsequent presentation *via* MHC-I or II molecules (53–56).

Next to CLRs, the Siglecs are also expressed on DCs and recognize self- and non-self-antigens (43, 46). Similar to CLRs, they could serve as adhesion molecules and endocytic receptors, and have been shown to be important instructors of T cell immunity (57, 58). Most members of the Siglec family signal through ITIM or ITIM-like motifs resulting in the generation of anti-inflammatory signals that modulate DC function (59). Overall, the ability of effective antigen uptake, processing, and presentation as well as the regulation of immunogenic and tolerogenic immune responses through the modulation of DC function positions DC receptors as promising candidates for novel DC-targeting immunotherapies. In the next sections, we explore current knowledge regarding the most promising DC-receptors being targeted for *in vivo* generation of tolDCs for the treatment of immune dysregulated pathologies.

## ITAM- and ITIM-Independent Receptors

### DEC205

**Figure 2:**
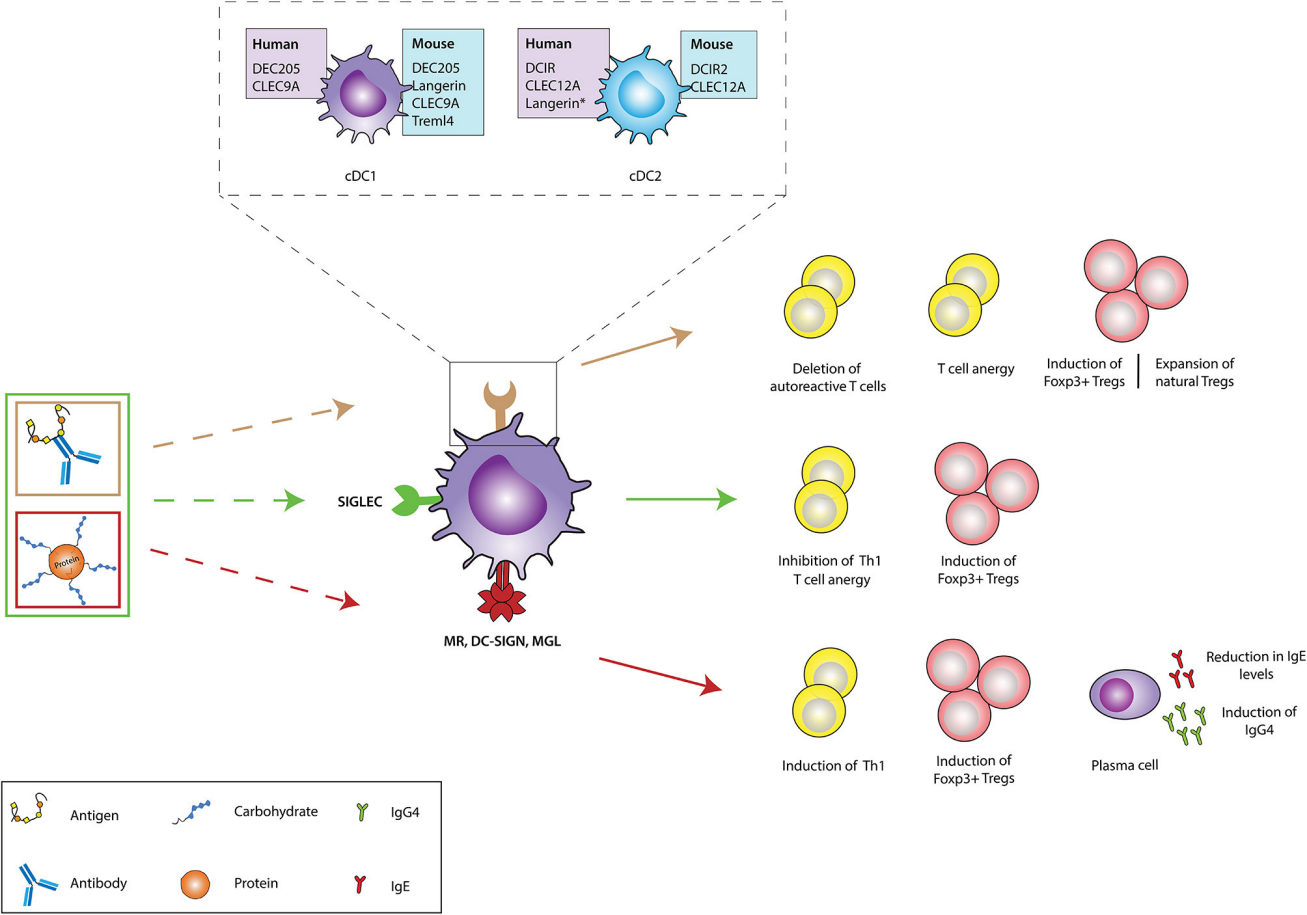
*In vivo* targeting of receptors on DCs against immunopathologies. Different receptors on dendritic cells (DCs) could be targeted using either antigen-antibody fusion compounds or carbohydrate-modified antigens. Brown arrows: the cDC1 and cDC2 subsets express a variety of receptors in both humans (purple boxes) and mice (blue boxes), which recognize and internalize fusion antibodies coupled with antigens, specific for a DC receptor and mediate the deletion of autoreactive T cells, induction of T cell anergy, generation of antigen-specific Foxp3^+^ Tregs and expansion of natural Tregs (These tolerogenic responses are abrogated if fusion antibodies are administered together with adjuvants like PolyI:C). The generation of either antigen-specific Foxp3^+^ Tregs or promotion of natural Tregs expansion depends of the receptor being targeted. Green arrows: Fusion antibodies against Siglecs, coupled with antigens or sialic acid-modified antigens bind to Siglec receptors on DCs and induce anti-inflammatory signals that result in the induction of Foxp3^+^ T cells, T cell anergy and the inhibition of Th1 responses. Red arrows: Targeting the MR, DC-SIGN and MGL on DCs with antigens conjugated to specific glycan moieties, result in the polarization of Th2 responses in allergy to Th1 responses and the induction of Foxp3^+^ T cells. Moreover, this interaction promotes the production of IgG4 blocking antibodies and mediates the reduction of IgE secretion. CLEC9A, C-type lectin domain family 9 member A; Treml4, The Triggering Receptor Expressed on Myeloid cells-like 4; DCIR, dendritic cell immunoreceptor; CLEC12A, C-type lectin domain family 12 member A; cDC1, Conventional type 1 dendritic cells; cDC2, Conventional type 2 dendritic cells; Siglec, Sialic acid-binding immunoglobulin-type lectins; MR, Mannose receptor; DC-SIGN, Dendritic Cell-Specific Intercellular adhesion molecule-3-Grabbing Non-integrin; MGL, macrophage galactose-type C-type lectin. Langerin*: Induced expression.

DEC205 (CD205) is an endocytic receptor highly expressed on cDC1s and belongs to the macrophage- mannose receptor family of CLRs (54). Although the natural ligand of DEC205 remains to be elucidated, some studies suggest apoptotic and necrotic material as well as CpG motifs as consecutive ligands (60). Upon antigen encounter, DEC205 internalizes and recycles very efficiently back to the surface (61). DEC205 was one of the first receptors used for *in vivo* antibody targeting of DCs (Table 1). Initial experiments using model antigens, such as hen egg lysozyme or ovalbumin (OVA), coupled to anti-DEC205 antibodies, demonstrated that these antigens were taken up by DCs (82–84). When OVA-anti-DEC205 fusion antibodies were administered to mice in the presence of maturation stimuli, strong immunogenic responses were induced (85). Conversely, when anti-DEC-antigens were injected into animals without adjuvants, DCs remained non-activated as their expression levels of costimulatory molecules was comparable to those obtained in DCs from control mice (83, 85). The analysis of antigen-specific T cell populations in injected mice revealed increased numbers of antigen-specific IL-10 producing CD25^+^Foxp3^+^ Tregs that were able to suppress proliferation of CD4^+^ T cells *in vivo* (83) (Figure 2). Since then, DEC205 targeting has been tested in various autoimmune disease animal models. For instance, in experimental autoimmune encephalomyelitis (EAE), the murine model for MS (63, 66, 86), injection of the autoantigen, myelin oligodendrocyte glycoprotein (MOG) or proteolipid protein (PLP) fused to anti-DEC205-specific antibodies led to elicitation of IL-10-producing CD4^+^CD25^+^Foxp3^+^ Tregs, the deletion of antigen-specific CD4^+^ and CD8^+^ T cells, reduced Th17 cell activity and significantly ameliorated disease symptoms and substantially delayed the disease onset (63, 64, 67) (Figure 2). In some studies, the conversion of some autoreactive T cells into Foxp3+ pTreg cells was reported. These findings were confirmed in non-obese diabetic (NOD), a mouse model of T1D, inflammatory bowel disease (IBD) (71), proteoglycan-arthritis (68), spontaneous experimental autoimmune uveoretinitis (EAU) (72), an animal model of ocular inflammation, as well as a model of graft-vs. host disease (65, 69, 70, 73). Because the use of DEC205 antibodies has proven to be more effective than the administration of free synthetic peptides, in these models of autoimmune diabetes, it is being considered as a possible, important therapeutic tool in the treatment of various autoimmune diseases (Table 1). In summary, these data establish DC targeting *via* DEC205 as an effective strategy to tolerize against autoantigens to protect against autoimmunity. However, this DC targeting strategy for the induction of tolerance is yet to be tested in human settings. Moreover, the future prospects of targeting DEC205 to induce tolerance in humans may be hampered by the varied expression pattern of this receptor on the different subsets of DCs (87). Although DEC-205 is predominantly expressed in mice CD8^+^ DCs, dermal DCs and LCs, human DEC-205 is relatively high expressed on myeloid blood DCs and monocytes, at moderate levels on B cells, and at low levels on pDCs, T cells and natural killer cells (87, 88). This differential expression pattern of DEC205 in humans is problematic for the development of DEC205-targeted vaccines for humans due to potential offsite targeting and needs to be addressed carefully during the design of potential clinical studies.

**Table 1:** Summary of *in vivo* studies to induce tolDCs using either antigen-antibody fusion compounds or carbohydrate-modified antigens.

**Receptor**	**Disease**	**Model**	**Antigen**	**Targeting strategy**	**Cellular response**	**Reference**
Clec9A		Multiple mouse models	OVA	Anti-clec9A	Foxp3^+^ T cells↑	(62)
DCIR2	MS	EAE mice	MOG	Anti-DCIR2	MOG-specific Foxp3^+^ T cells	(63)
			PLP	Anti-DCIR2	CD4^+^Foxp3^+^ T cells↑, pathogenic T cells↓	(64)
	T1D	NOD mice	BDC2.5	Chimeric anti-DEC205 (33D1)	CD4^+^Foxp3^+^ T cells↑, T cell apoptosis	(65)
DEC205 (CD205)	MS	EAE mice	MOG	Anti-DEC205, single-chain fragment variables specific for DEC205	CD4^+^CD25^+^Foxp3^+^ T cells↑	(63, 66)
			PLP	Chimeric anti-DEC205	CD4^+^Foxp3^+^ T cells↑, CD4^+^ Th17 cells↓	(64, 67)
	RA	PGIA mice	PG	Anti-DEC205	CD4^+^Foxp3^+^ T cells↑, PG-specific CD4^+^ cells↓	(68)
	T1D	INS-HA/TCR-HA transgenic mice	HA	Anti-DEC205	CD4^+^Foxp3^+^ T cells↑, CD4^+^CTLA4^+^ T cells↑	(69)
		NOD mice	BDC2.5, MimA2	Chimeric anti-DEC205	Antigen-specific CD4^+^ and CD8^+^ T cells↓	(65, 70)
	IBD	VILLIN-HA transgenic mice	HA	Anti-DEC205	HA-specific CD4^+^Foxp3^+^ T cells↑	(71)
	EAU	Spontaneous EAU mice	HEL	Anti-DEC205-HEL	CD4^+^CD25^+^Foxp3^+^ T cells↑, CD4^+^ cells↓	(72)
	GVHD	C57BL/6 mice	hNC16A	Anti-DEC205-hNC16A	CD4^+^ and CD8^+^ graft infiltration↓	(73)
Langerin (CD207)	MS	EAE mice	MOG	Anti-Langerin	Foxp3^+^ T cells↑	(63)
MR, DC-SIGN, MGL	MS	EAE mice	PLP	Mannosylation	T cell proliferation↓	(74, 75)
	Grass pollen allergy	Human cells, BALB/c mice	Polymerized *P. pratense* allergens	Mannosylation	CD4^+^CD25^+^Foxp3^+^ T cells↑	(76, 77)
	Birch pollen allergy	Human cells, BALB/c mice	Bet v 1	GalNAc-linking, mannosylation	T cell proliferation↑	(78)
Siglecs	MS	EAE mice, BALB/c mice	MOG, OVA	Chimeric anti-Siglec-H	CD4^+^ T cell anergy	(79)
		C57BL/6 mice	OVA	Sialyation	CD4^+^Foxp3^+^ T cells↑	(80)
	Grass pollen allergy	BALC/c mice	Phl-p5a	Sialylation	CD4^+^Foxp3^+^ T cells↑, CD4^+^ Th2 cells↓, eosinophilic airway inflammation↓	(81)
Treml4	MS	EAE mice	MOG	Anti-Treml4	MOG-specific Foxp3^+^ T cells↑	(63)

*EAE, experimental autoimmune encephalomyelitis; EAU, experimental autoimmune uveoretinitis; GVHD, graft versus host disease; HA, hemagglutinin; IBD, inflammatory bowel disease; MimA2, mimotype peptide; MOG, myelin oligodendrocyte glycoprotein; MS, multiple sclerosis; NOD, non-obese diabetic; OVA, ovalbumin; PG, proteoglycan; PLP, proteolipid protein; RA, rheumatoid arthritis*.

### DC-SIGN, MR, and MGL

DC-SIGN and MR are other members of the CLR family which recognize several mannose and fucose-containing structures, present on many antigens (49) and activate signaling pathways in CLR-expressing cells (44, 52, 60). These receptors are widely expressed on DCs and have been extensively exploited in several fields as potential targets for immunotherapy (89–91). Previously, antibody-mediated CLR targeting has been the most studied strategy for antigen delivery and activation of DCs *in vivo*, but in recent years glycan-based targeting approaches are gaining increasing attention (Table 1) (89, 90). Compared to antibody-mediated targeting, in glycan-based targeting, the spatial orientation of displayed carbohydrate CLR ligands can be varied more easily according to the distances between receptor binding sites thereby enhancing receptor-ligand binding and subsequent signaling (89). DC-SIGN is exclusively expressed on immature DCs and shows properties that are often, but not always, associated with Th2 polarization, suppression of inflammation and/or induction of regulatory immune response inhibiting pro-inflammatory Th1/Th17 immunity, especially when it recognizes helminth or allergen associated antigens (92–96). Interestingly, binding of the mycobacterial cell wall component Mannose-capped Lipoarabinomannan (ManLAM) to DC-SIGN inhibits DC maturation and induces IL-10 production (97). Also, the use of fucosylated ligands targeting DC-SIGN biases immune responses toward anti-Th1 responses, with an enhanced Th2 response, and has been shown to ameliorate different autoimmune conditions pre-clinically (92, 98). For instance, exposure of NOD mice to fucose-containing schistosome antigens inhibited the development of type 1 diabetes. This finding is in agreement with reports that have shown that such glycan-CLR signaling can induce a regulatory T cell phenotype having IL-10 and TGF-β production (99), which could explain the observed prevention of the development of autoimmunity in these mice (98, 100).

The MR recognizes terminal mannose, fucose and *N*-acetylglycosamine carbohydrates *via* its carbohydrate recognition domains. In humans, the MR has been identified in CD1a^high^ and CD1a^low^ dermal DCs, as well as *in vitro* monocyte-derived DCs and macrophages (101). In mice, the MR is mainly expressed by tissue and lymphoid-resident macrophages, but also in various endothelial cells and tracheal smooth muscle cells (101, 102). Additionally, MR expression can be detected in cultured murine moDCs, however the *in vivo* expression of MR on murine DCs remains unknown (61, 101). The MR has been reported to induce DC-mediated anti-inflammatory responses, including IL-10 production upon binding to some natural ligands that bind inside the MR binding sites (103). In contrast, when MR interacts with ligands that bind outside the carbohydrate recognition domains, there is no induction of IL-10 secretion, suggesting that, the efficacy of MR-targeted vaccines to induce tolerance will greatly depend on the appropriate selection of targeting vehicles and conditions (104). Notably, in a murine autoimmune model of collagen antibody-induced arthritis, treatment of mice with an epitope of *Leishmania* analog of the receptors for activated C kinase (LACK) from *Leishmania major*, inhibited joint inflammation and downregulated Th1 and Th17 cell responses through binding to the MR in CD11c^+^ DCs (105). Similarly, mannosylated forms of the myelin peptide PLP_139−151_ and MOG induced a state of tolerance in EAE mice (74, 75). Inhibition of EAE disease severity was suggested to be mediated by modulation of peripheral autoreactive T cells. This is in agreement with a study where treatment with mannosylated OVA peptides induced impaired Th1 effector functions and abrogated the activity of pre-existing effector T cells (106).

MGL is well-characterized for its specificity for terminal GalNAc (N-Acetylgalactosamine) residues, expressed by both mammalian cells and pathogens (49). In humans, MGL is expressed *in vivo* by human DCs of skin and lymph nodes and *in vitro* by macrophages and moDCs (107). In mice however, the homologs of human MGL, MGL1, and MGL2 are expressed by dermal DCs and alternatively activated macrophages. Upon ligand binding, the intracellular signaling pathways that are triggered vary extensively depending on the structure of the ligands. In this regard, it has recently been reported that, glycoconjugates from *Fasciola hepatica* potentiate the production of IL-10 by moDCs *via* engagement of MGL (94). Moreover, MGL-expressing DCs from mice infected with these glycoconjugates expanded IL-10-producing T cells and suppressed Th1 responses. Correspondingly, recent data has labeled the MGL as a negative regulator in autoimmune-induced neuroinflammation as MGL was shown to induce apoptosis of autoreactive T cells, the reduction of autoantibodies and the induction of IL-10 (108).

The use of glycan-based strategies has been substantially tested in pre-clinical and a few clinical settings for the treatment of allergies (Table 1). In these studies, carbohydrate-modified allergens were used to dampen allergic immune responses while installing antigen-specific T cell anergy both *in vivo* and *in vitro* (76, 77, 109–112). A notable mention is a study by Sirvent *et al* where they conjugated non-oxidized mannan from *Saccharomyces cerevisae* to polymerized grass pollen allergens (PM) and demonstrated that PM-treated human moDCs favor the induction of CD4^+^CD25^high^CD127^−^Foxp3^+^ Tregs over Th1 cells through PD-L1 signaling, subsequently causing an increase in the IL-10/IL-5 cytokine ratio produced by T cells (76) (Figure 2). PM was captured *via* the MR and DC-SIGN and proved to be hypoallergenic during *in vivo* skin prick tests and *ex vivo* basophil activation tests. The same group demonstrated that this strategy is equally effective in the treatment of canine atopic dermatitis (77). Interestingly, oxidation of mannan impaired the tolerogenic properties of PM shown in both human and mice, emphasizing the importance of the mannan structure for its functional properties (76, 109, 113). Also, Mathiesen et al. used the major birch pollen allergen, Bet v 1, coupled to defined carbohydrate structures and demonstrated that the prophylactic treatment of mice with GalNAc-coupled Bet v 1 significantly reduces IgE responses (78) (Figure 2). This finding suggests that MGL, which recognizes terminal α-and β-linked GalNAc structures, might be involved in the induction of the observed immune responses and may thus qualify as another potential candidate for specific antigen-delivery to DCs for induction of tolerance (114). Cumulatively, available data suggest that targeting DC-SIGN, MR and MGL for specific antigen delivery is a promising strategy to be further explored for the management of dysregulated immune pathologies (Figure 2).

### Langerin

Langerin (CD207) is a transmembrane protein that functions as an endocytic receptor by binding various sugars, including mannose, *n*-acetylglucosamine, fucose, and sulfated sugars, and mediates efficient antigen presentation on MHC I and II products *in vivo* (115). Langerin is highly expressed on surfaces of human LCs, but also at low levels on cDC2s isolated from dermal, lung, liver and lymphoid tissue (116). Yet, langerin is not expressed on circulating cDC2s isolated from blood (116). In mice, langerin is expressed on LCs and CD8α^+^DEC205^+^ cDC1s of the spleen and skin draining, however, langerin has not been identified in the homologous human cDC1 subset, indicating that langerin targeting strategies could induce distinct outcomes between mice and human experiments due to differential expression of the targeting receptor in both species (44, 52). Nevertheless, the tolerogenic role of LCs under physiological conditions as well as their accessible location at the surface of the body, marked langerin as a promising target for *in vivo* delivery of self-antigens to alter disease severity in autoimmune diseases (30, 31, 63). A notable mention is a report from Idoyaga *et al* that showed that, targeting MOG_35−55_ peptides to murine skin and lung langerin^+^ migratory DCs *via* conjugation with anti-langerin antibodies lessens EAE symptom severity through the induction of Foxp3^+^ Tregs (63) (Figure 2). The effect was comparable to the reduction of disease symptoms following administration of anti-DEC205-MOG fusion proteins. Interestingly, the langerin^+^ DC population is known to co-express high levels of DEC205, suggesting that the DC subset that is targeted with anti-langerin is also a target for anti-DEC205 mediated induction of Foxp3^+^ Tregs (117). However, in contrast to the above findings, co-administration of recombinant langerin-mAbs fused to antigen and maturation stimuli like anti-CD40 or polyI:C leads to efficient CD4^+^ and CD8^+^ T cell priming, proliferation, and differentiation (118). These results suggest that the strength of the activation signal targeted to langerin^+^ DC is a very important factor that must be strictly controlled in order to exploit these subsets in autoimmune therapy. Nonetheless, the ability of langerin^+^ DCs to induce antigen-specific Foxp3^+^ Tregs in lungs, suggest that anti-langerin mAbs is an attractive candidate for the treatment of respiratory dysregulated immune responses like allergies (Figure 2).

### Cell Death Receptors: Clec9A and Treml4

DCs are able to recognize and take up DAMPs through surface receptors such as Clec9A, a homodimeric type II transmembrane protein with a single extracellular C-type lectin-like domain expressed on cDC1s in both mice and human (119–121). The highly restricted expression of Clec9A on the human and mice cDC1 subset makes it an attractive receptor for targeting this specific subset of DCs (118). Clec9A ligation to its ligand F-actin either results in immunity or tolerance. As Clec9A promotes CD8^+^ T cell cross-priming, several *in vitro* studies have been performed to explore Clec9A targeting to induce anti-tumor immune responses (121). To determine whether Clec9A is a promising receptor for DC targeting in the context of autoimmune diseases, mice were injected with anti-Clec9A-antigen conjugates. In steady-state conditions in the absence of adjuvants, these conjugates promoted the differentiation of Foxp3^+^ Treg cells (62) (Figure 2). On the other hand, when anti-Clec9A was administered in combination with polyI:C, tolerance was prevented and instead promoted the development of potent antibody and Th1 or Th17 responses (62). Also, it has been reported that antigen delivery *via* Clec9A enhances the humoral response, even in the absence of adjuvant CpG. However, the immunoglobulin classes and resulting tolerogenic or immunogenic functions, were not explored in this study (122). Although targeting Clec9A can induce Tregs, extensive research is still needed to perfectly map out the optimal conditions that are necessary for tolerance induction.

Treml4 is another cell death receptor and binds to late apoptotic bodies necrotic cells (123). It is a member of the the triggering receptor expressed on myeloid cells (Trem)-family receptors which are primarily expressed on murine CD8^+^ lymphoid resident DCs and CD103^+^ lung DCs (63, 123). Treml4 has been investigated as a therapeutic target in a study by Idoyaga and colleagues. In this study, it was demonstrated that, intranasal inoculation of anti-Treml4-MOG peptide conjugates could induce MOG-specific Foxp3^+^ T cells in mice, but this did not prevent the development or promote improvement of EAE symptoms in diseased mice (63). The molecular mechanisms underlying these observations were not explored but it seems like signaling through this receptor does not produce a strong enough signal necessary for DC-mediated polarization of T cells to Tregs or the suppressive capacity of induced Tregs may be impaired in some way. Mechanistic studies addressing these issues will be very valuable in further exploring the therapeutic potential of this receptor in human settings in the context of immune pathologies. It is also important to note that the expression of Treml4 on human DC is yet to be reported. Therefore, the use of cell death sensing receptors for tolerizing therapies remains elusive until additional studies shed light on their relevance for clinical applications.

## ITIM-Bearing Receptors

### The Siglec-Family

Siglec receptors are a family of receptors expressed on a wide variety of immune cells, including DCs (43, 59). They recognize sialic acid, which is the last carbohydrate structure added during the process of glycosylation, positioning sialic acid groups on the distal end of sugar-moieties (124). These sialic acid groups are present in the glycocalyx of all mammalian cells and could be considered as SAMPs (43). Siglecs are divided into two groups: (1) Siglecs that are conserved throughout different species, namely Siglec-1 (sialoadhesin), Siglec-2 (CD22), Siglec-4 myelin associated glycoprotein (MAG) and Siglec-15, and (2) the CD33-related Siglecs that have rapidly expanded and have no clear orthologs in mammalian species viz; Siglec−3 (CD33), −5, −6, −7, −8, −9, −10, −11, −14 and −16. The expression of Siglecs on myeoloid cells and their subsequent intracellular signaling pathways have been nicely reviewd by Lübbers et al. (59). In short, monocytes and monocyte-derived DCs express high levels of Siglec-3, −7 and −9, and low levels of Siglec-10. cDCs also express Siglec-3, −7, and −9, augmented with low expression levels of Siglec-2 and −15. On the other hand, pDCs only express Siglec-1, which is a non-signaling Siglec that internalizes upon ligand binding, and Siglec-5 (59). The binding affinity to sialic-acid-containing glycan varies between Siglecs. This is determined by the linkage of the sialic acid group to the underlying carbohydrate moiety (α2,3; α2,6 or α2,8 linkage). Another noteworthy feature of Siglecs is their ability to either bind their ligand *via* a *trans* interaction (on a different cell) or *via* a *cis* interaction (on the same cell). These *cis* interactions might contribute to sustain a tolerogenic phenotype, as surface proteins on tolerogenic DCs, immature DCs and Tregs are highly α2,6-sialylated, and could serve as a ligand for tolerogenic Siglecs (125). The conserved Siglec-2 and the CD33-related Siglec-3, and−5 till−11 contain an intracellular ITIM or ITIM-like motif that deliver negative signals *via* recruitment of SHP1 and SHP2 (43).

Interestingly, this immune modulating mechanism has been exploited by pathogen and cancer cells. For example, the protozoan parasite *Trypanosoma cruzi* enzymatically cleaves sialic acid moieties from the host and transfers them *via* α2,3-linkage to its own surface, subsequently downregulating pro-inflammatory IL-12 production and upregulating anti-inflammatory IL-10 production in murine DCs (126). Furthermore, various tumors upregulate α2,3; α2,6; and α2,8 sialic acid on their surface to evade anti-tumor T cell responses and to induce tumor-specific tolerance (59). Comparable to these natural Siglec-mediated immune modulation events, the tolerance inducing capacity of Siglecs could be used in therapeutic strategies to treat auto-immune and allergic diseases. So far, several *in vitro* and *in vivo* mouse studies have been performed to address this concept (Table 1). Targeting Siglec H on murine pDCs using anti-Siglec-H-antigen (OVA or MOG peptides) conjugates resulted in a decrease of CD4^+^ T cell expansion and Th1/Th17 differentiation, which subsequently delayed the onset and reduced disease severity in EAE when using the anti-Siglec-H-MOG conjugate (79). Similarly, direct modification of OVA and MOG peptides with α2,3 or α2,6 sialyl-lactose targeted these antigens to Siglec E on DCs and dampened pro-inflammatory responses in the same EAE mouse model upon treatment with sialylated MOG peptides (80). Siglec E targeting resulted in the induction of Foxp3^+^ CD4^+^ Tregs and inhibition of inflammatory effector cells after stimulation with LPS, both *in vitro* and *in vivo* (80) (Figure 2). Finally, the potential of sialic acid modified antigens was tested in an experimental murine model for grass pollen allergy. Subcutaneous treatment with sialic acid modified grass pollen peptides induced significant numbers of antigen specific Tregs, inhibited antigen specific effect Th2 cells, and reduced the accumulation of eosinophils (81).

Overall, these studies demonstrate that targeting DC through Siglecs could be very promising for induction of tolerogenic immune responses as a treatment for autoimmune and allergic diseases (Figure 2). However, most studies have been performed in mice and are therefore not sufficient for translation to human settings. Consequently, it is important to elucidate their potential and consequences in the human immune system.

### DCIR

DCIR is another member of the family of CLRs expressed on cDCs, moDCs and pDCs (44, 45, 127). The human DCIR-Fc protein has been reported to bind a variety of carbohydrate structures including Lewis^b^, Man3 glycans, and bisecting GlcNAc residues (127). The mouse homolog of DCIR, DCIR2 is primarily expressed on CD8^+^DCs. DCIR is important for the homeostasis of the immune system by, in part, regulating DC differentiation or polarization, as DCIR-deficient mice were prone to develop autoimmune encephalitis (128). As such, consistent with results obtained in mouse models (128, 129), polymorphisms of the DCIR gene are associated with the susceptibility to RA in humans (130). DCIR2^+^ DCs have been shown to stimulate natural Foxp3^+^ Tregs to mediate tolerance to self-antigens in the absence of immune stimuli (41). However, in the presence of a maturation stimulus, they induce T cell expansion and production of pro-inflammatory cytokines (41). Upon triggering with DCIR-specific mAbs, DCIR is internalized in both pDCs as well as human moDCs, resulting in efficient antigen presentation to T cells and downregulation of IFN-α production (131). Interestingly, using an anti-DCIR2-PLP_139−151_ and MOG fusion antibody to target DCs resulted in the amelioration of EAE symptoms (63, 64). This effect was suggested to be mediated primarily through the depletion of autoreactive T cells or induction of anergy in pathogenic T cells (Figure 2). However, DCIR2-targeting did not induce *de novo* generation of Ag-specific Treg from naïve CD4^+^ T cell precursors in the steady state due to lack of TGF-β expression by CD4^+^ CD11b^+^ DC but instead stimulated and expanded natural Tregs (64). Similarly, targeting β-Cell antigen using chimeric DCIR2 antibodies in NOD mice, elicited tolerogenic CD4^+^ T cell responses and induced increased T cell apoptosis while delaying diabetes induction (65). Targeting DCs with anti-DCIR2-antigen has also shown some promise in the field of transplantation where, an anti-DCIR2-MHC I monomer successfully inhibited allorecognition and the production of IgG alloantibodies leading to long-term allograft survival (132). Unexpectedly, DCIR2 targeting of mice DCs augmented spontaneous EAU development, characterized by local reduction in Tregs (133). While DCIR2 may be a promising candidate for *in vivo* Ag delivery in mice, this may not be the case for humans because, even though DCIR2 is highly restricted to CD11b^+^ DC subset in mice it is not detectable on the human CD1a/b^+^ cDC subset, a proposed equivalent of mouse CD11b^+^ DCs (130). Thus, there is need for identification of surface molecules that are specifically and similarly expressed by mouse and human DCs to allow exploration of clinical effectiveness of vaccines targeting these DC subsets.

### Clec12A (MICL)

The human myeloid inhibitory C-type lectin receptor (MICL) or Clec12A is expressed on alveolar macrophages, cDC1s, cDC2s, and pDCs, while the mouse Clec12A is expressed on myeloid cells (45, 134). Clec12A selectively binds to dead cells that have lost their plasma membrane integrity (130). The endocytic capacity of Clec12A has led to its being exploited for DC-specific antigen targeting. In this regard, antibody-mediated targeting of OVA to Clec12A in mice was able to induce potent antibody responses but no tolerogenic responses (135). Such targeting of Clec12A with anti-Clec12A antibodies seems to be sufficient for antigen internalization, processing and presentation but not for activation of DCs as reported in targeting of DEC205 with mAbs (122, 135). These mAbs may therefore simply serve to deliver Ags to DCs. The study of Clec12A in the context of immunotherapy for dysregulated immune pathologies is still in its infancy and further research is warranted given that preliminary data and the biological properties of Clec12A portrays this receptor as a promising candidate in this field.

## Future Perspectives and Conclusions

Due to the unique capacity of DCs to coordinate innate and adaptive immune responses, they have been extensively studied and have proven to be a very promising strategy for immunotherapy. In the past decades, our knowledge of the potential of DCs in cancer and autoimmune disease/allergy therapy has expanded remarkably, advancing from the current *ex vivo* generated DC-based vaccines to *in vivo* targeting of DCs *via* specific receptors (Figure 1). Various compounds, such as vitamin D3, retinoic acid, dexamethasone, or IL-10, and TGFβ have shown the potency of tolDCs as immunotherapy in autoimmune diseases. However, there has been an increasing interest in moving toward *in vivo* targeting strategies where the induction of tolerance is achieved by targeting different receptors on DCs in their natural environment with antigen-delivering antibodies and antigen-carbohydrate conjugates. This has proven to be very effective in the amelioration of disease processes in a range of mouse models including MS, diabetes and allergies. Nevertheless, there is still a need to expand our knowledge on the potential application of such *in vivo* targeting strategies in human settings because, despite the many important similarities that exist between human and mouse DCs, very crucial incompatibilities between both species still limits the capacity to translate findings from one species to the other. Nonetheless, the potential for future clinical translation and therapeutic application of *in vivo* antigen targeting to DCs is very promising, although additional research is necessary to decipher the specific molecular mechanisms involved in the anti-disease tolerance promoted by such DCs. During the development of potential vaccines for autoimmune and allergic diseases, multiple inevitable questions need to be addressed. For instance, receptors that are not inherently tolerogenic but are capable of inducing tolerance under certain conditions, such as DEC205, DC-SIGN, and langerin, the induced tolerogenic effect is abrogated in the presence of pro-inflammatory modulators. Therefore, the appropriate optimization of vaccine formulations to target such receptors will be of utmost importance. In contrast, Siglecs have the ability to induce tolerogenic immune responses even in the presence of the pro-inflammatory modulator LPS (80, 136) and the resulting responses are not particularly affected by the presence of adjuvants. Moreover, there is still uncertainty about the right antigen-antibody/glycan dosage necessary for induction of tolerance, the duration of the resulting tolerogenic response, the effect on other immune cells expressing similar receptors as those being targeted, the use of a vehicle, and the method of administration. Finally, it may also be important to further investigate the potential positive or negative effects that receptor-specific antigen targeting may have on other myeloid cells, such as macrophages that express some of the DC receptors that can be targeted. Although further investigation is warranted, the effects might be negligible giving the lower antigen presenting capacity of macrophages. Overall, it is clear that the generation of a tolerogenic immune response *via* DC receptor targeting depends on the receptor, the DC subset being targeted, and the specific micro-environmental factors.

## Author Contributions

CC and B-CKD performed the literature search, wrote the manuscript, and created all figures. EdJ and YvK critically read and carefully revised all versions of the manuscript providing valuable guidance and insight. RvR critically read the manuscript and provided valuable additions. All authors contributed to the article and approved the submitted version.

## Conflict of Interest

The authors declare that the research was conducted in the absence of any commercial or financial relationships that could be construed as a potential conflict of interest.
